# Urinary liver-type fatty acid-binding protein variation as a predictive value of short-term mortality in intensive care unit patients

**DOI:** 10.1080/0886022X.2021.1943439

**Published:** 2021-06-30

**Authors:** Ginga Suzuki, Ryo Ichibayashi, Saki Yamamoto, Hibiki Serizawa, Yoshimi Nakamichi, Masayuki Watanabe, Mitsuru Honda

**Affiliations:** Critical Care Center, Toho University Omori Medical Center, Tokyo, Japan

**Keywords:** Critical care, critically ill, ICU, intensive care, L-FABP, liver-type fatty acid-binding protein

## Abstract

**Background:**

Predicting the prognosis of intensive care unit (ICU) patients is crucial because it may lead to patient stratification that would in turn help in appropriately distributing limited medical resources. This study, therefore, aimed to investigate the use of the urinary liver-type fatty acid-binding protein (L-FABP) semi-quantitative kit in rapidly predicting the prognosis of patients admitted to the ICU.

**Methods:**

We conducted a single-center, prospective, observational study wherein 100 consecutive patients admitted to the ICU with an indwelling bladder catheter were enrolled between April and October 2020. Urine specimens were collected at the time of admission (T1) and after 6 h (T2), and urinary L-FABP levels were semi-quantitatively measured. Based on the results, an L-FABP variation was defined as the change in L-FABP (negative, weakly positive, or strongly positive) from T1 to T2. Patients were divided into three groups (L-FABP decreased group, unchanged group, or increased group), following which we compared their 14-day mortality.

**Results:**

Finally, a total of 79 patients were included in the analysis. In multivariate analysis, urinary L-FABP variation [Odds ratio (OR) = 14.327, 95% confidence interval (CI) = 1.819–112.868, *p* = 0.01] and lactate (OR = 1.234, 95%CI = 1.060–1.437, *p* = 0.01) were significantly associated with 14-day mortality.

**Conclusion:**

Urinary L-FABP variation at 6 h after admission was significantly associated with 14-day mortality.

## Background

The mission of intensive care units (ICUs) is not limited to intensive care, which is a medical and surgical treatment but also includes properly distributing limited medical resources. This can be achieved by predicting the prognosis of patients in the ICU as it may allow for patient stratification and the appropriate distribution of medical resources. Additionally, clarifying confounding factors associated with prognosis can provide biases to consider when conducting future studies.

Liver-type fatty acid-binding protein (L-FABP) is expressed in the liver as well as in the intestine, pancreas, stomach, lung, and kidney. L-FABP has a high affinity and binding capacity for fatty acid peroxidation products and causes their excretion into urine, and thus may be an effective endogenous antioxidant [1].

We previously reported a semi-quantitative measurement kit for urinary L-FABP that can predict the onset of acute kidney injury (AKI) after admission to the ICU [2]. We found that urinary L-FABP-positive cases were significantly associated with AKI development when compared to L-FABP-negative cases; however, there was no such association observed with mortality. In contrast, urinary L-FABP was reported to predict AKI as a renal ischemia marker from an early stage [3,4]. Contrary to our previous findings, Doi et al. reported an association between urinary L-FABP and 14-day mortality, in addition to the onset of AKI [5]. L-FABP was originally discovered as a specific marker for AKI; however, in recent years, it has been reported that urinary L-FABP increases due to damage to various other organs or surgical invasion [6–14], and therefore, it has also emerged as a marker of general organ damage.

Therefore, we hypothesized that the prognosis of ICU patients could be predicted using the urinary L-FABP semi-quantitative kit. First, if urinary L-FABP is indeed associated with the prognosis, our previous results suggest that it should remain higher than the positive cutoff of the kit. Considering this approach, a change in a patient’s urinary L-FABP—from positive to negative—would indicate an improvement in their condition; we believe that this, in turn, signifies a good outcome. Therefore, we investigated whether the prognosis of ICU patients could be predicted using variations in urinary L-FABP levels. This study is an exploratory study that aims to investigate the possibility of a simple and rapid prediction of prognosis using the urinary L-FABP semi-quantitative kit among ICU patients.

## Methods

### Design and setting

This single-center, prospective, and observational study was conducted at the Critical Care Center, Toho University Medical Center Omori Hospital. This study was conducted by the Declaration of Helsinki and was approved by the Ethics Committee of Toho University Omori Medical Center (approval number M17084). All participants had provided written informed consent. If a patient could not provide consent, a consent form was obtained from the patient's family (an adult family living together or a relative within the third degree of kinship). This study is observational and is reported by the Strengthening the Reporting of Observational Studies in Epidemiology statement.

### Subjects

Patients who were transferred to the ICU between April and October 2020 from tertiary emergency care were included. Of these, 100 consecutive patients who were admitted to the ICU with an indwelling bladder catheter were enrolled. To avoid taking urine samples just for research purposes, we included patients who were admitted to the ICU with an indwelling bladder catheter that was deemed therapeutically necessary. Since this is an ICU for post-operative patients, tertiary emergency patients, and inpatients with acute deterioration, most of the ICU patients have an indwelling bladder catheter. Since this was an exploratory study, the sample size was not calculated. However, since the sample size of a previous study that used the same kit was 250 [2], we estimated the need for approximately half of the sample size of the previous study as we performed two-point measurements in this study. The exclusion criteria were as follows: age <18 years, death within 6 h of hospitalization, maintenance dialysis, historical kidney transplantation, and performed emergency surgery. Patients undergoing maintenance dialysis and those with a history of renal transplantation were excluded because those factors were considered to have a strong influence on urinary L-FABP levels. Patients requiring surgery were excluded because surgical invasion may also affect urinary L-FABP levels. Patients who died within 6 h were excluded because we observed changes in urinary L-FABP levels at 6 h following admission.

### Measurements

Urine specimens were collected at the time of admission (T1) and after 6 h of admission (T2), and urinary L-FABP levels were measured. A rapid semi-quantitative assay kit, RENAPRO^®^ (CMIC Pharmaceutical Services Co., Ltd., Tokyo, Japan) was used for the measurement of urinary L-FABP levels. The kit determines that an L-FABP level <12.5 ng/ml is negative, ≥12.5 and <100 ng/ml is weakly positive, and ≥100 ng/ml is strongly positive. In negative cases, the judgment line is not displayed in the test column whereas weak and strong positives are indicated by light and dark lines, respectively. Urine volume after admission was measured hourly using a urinary catheter, and all samples contained urine excreted within 1 h of collection. The nurse in charge collected urine, mixed it immediately with the reagent, and started the measurement. The results were obtained digitally and captured in an electronic medical record.

### Data collection

Data including patient profiles, physical findings, examination data, and urinary L-FABP results were registered in our data resources and electronic medical records. After including 100 patients, we extracted data from electronic medical records from November to December 2020.

The following items were extracted: age; sex; height; weight; body mass index (BMI); primary disease; Charlson comorbidity index (CCI) [15]; C-reactive protein (CRP); albumin (Alb); blood urea nitrogen (BUN); creatinine (Cr); lactate; white blood cell count; hemoglobin; Acute Physiology and Chronic Health Evaluation (APACHE) II score; onset of AKI after admission (diagnosed when the criteria of stage 1 or higher are met based on the Kidney Disease: Improving Global Outcomes guidelines) and finally, urinary L-FABP determination (T1 and T2 negative/weakly positive/strongly positive. The semi-quantitative results of urinary L-FABP were judged by a single physician (G.S.), independent of treatment. In addition, to evaluate the intra-rater reliability, the results of all urinary L-FABP of T1 were reevaluated and recorded one week later. The second judgment was used to evaluate the reliability alone.

The urinary L-FABP semi-quantitative concentration between T1 and T2 decreased (decreased group), remained unchanged (unchanged group), or increased (increased group).

### Outcomes

The primary endpoint was 14-day mortality. The secondary endpoints were 28-day mortality and length of ICU stay. Length of ICU stay was calculated from the date of admission to that of death in ICU or discharge from ICU.

### Statistical analysis

The intra-rater reliability in judging the results of urinary L-FABP was evaluated by the intraclass correlation coefficient (ICC) and the kappa coefficient [16,17].

Continuous variables not rejected by the Kolmogorov–Smirnov test (normal distribution) are expressed as mean ± SD, and those rejected (not normal distribution) are expressed as median (interquartile range), while categorical and ordinal variables are represented as percentages. The one-way analysis of variance in normal distribution and the Kruskal–Wallis test is not normal distribution was used for continuous variables, and the χ-square test was used for categorical and ordinal variables. The outcomes were compared after comparing the background factors of each group.

First, to clarify the significance of performing the two-point measurement, the results of L-FABP at the time of admission and 6 h after admission were analyzed. A univariate logistic regression analysis was performed with the 14-day mortality rate as the objective variable and each background factor and L-FABP at admission as the explanatory variable. Separately, a similar univariate analysis was performed by substituting L-FABP 6 h later instead of L-FABP at admission. Next, a multivariate logistic regression analysis was performed with a 14-day mortality rate as the objective variable, age, sex, primary disease, CCI, factors reported to be associated with prognosis, factors found to be statistically significant in univariate analysis, APACHE II score, AKI, and urinary L-FABP at admission and 6 h later as the explanatory variable.

We investigated whether variations in urinary L-FABP (decreased/unchanged/increased) were associated with 14-day mortality. A multivariate logistic regression analysis was performed with a 14-day mortality rate as the objective variable, age, sex, primary disease, CCI, factors reported to be associated with prognosis, factors found to be statistically significant in univariate analysis, APACHE II score, AKI, and urinary L-FABP variation as the explanatory variable. Factors that have been reported in previous studies are BMI, CRP/Alb ratio, and lactate level [18–20].

When there were many explanatory variables, multivariate analysis using propensity scores was performed for sensitivity analysis. Of the explanatory variables used in the above multivariate analysis, variables other than L-FABP variation were used to calculate the propensity score for 14-day mortality by logistic regression. Finally, the receiver operating characteristic (ROC) curve of L-FABP variation for 14-day mortality was drawn and the area under the curve (AUC) was calculated.

Statistical analyses were performed using StatFlex^®^ version 7 (Artec, Osaka, Japan). When the ICC and kappa coefficient was >0.8, it was judged that the intra-rater reliability was high [16,17]. Differences were considered statistically significant when the *p*-value was <0.05.

## Results

**Figure 1.**
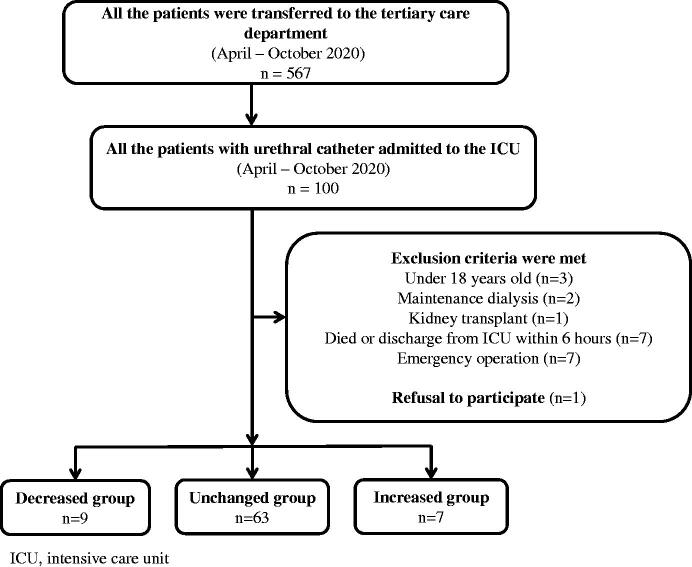
Flow chart of patient selection. ICU: intensive care unit.

We enrolled a total of 100 participants in the study. According to the exclusion criteria, we excluded three patients under the age of 18 years, two under maintenance dialysis, one patient who had undergone kidney transplantation, seven who died or were transferred within 6 h, and seven who required surgery. Patients who were pregnant, monorenal, or with known chronic kidney injury were excluded. In addition, one patient was excluded due to a lack of consent. Finally, a total of 21 patients were excluded, and 79 patients were included in the analysis. There were nine patients in the decreased group, 63 in the unchanged group, and seven in the increased group (Figure 1).

There were no significant differences in any background factors among the three groups (Table 1). In particular, lactate and creatinine levels were not significantly associated with L-FABP variation. There was no correlation between basal GFR and L-FABP on admission (data not shown). There was also no correlation between the APACHE II score and L-FABP variation (data not shown).

**Table 1.** Baseline characteristics.

Parameters	Decreased group (*n* = 9)	Unchanged group (*n* = 63)	Increased group (*n* = 7)	*p*-Value
Age, years	72.8 ± 11.0	62.4 ± 19.4	67.4 ± 17.6	0.29
Male, *n* (%)	7 (77.8%)	41 (65.1%)	3 (42.9%)	0.34
Height, m	1.7 ± 0.1	1.6 ± 0.1	1.6 ± 0.1	0.37
Weight, kg	57.7 ± 11.9	58.2 ± 12.0	61.6 ± 27.5	0.83
Body mass index, kg/m^2^	21.0 ± 3.4	22.1 ± 3.8	23.4 ± 7.9	0.66
Primary disease				0.84
Cardiovascular, *n* (%)	2 (22.2%)	9 (14.3%)	0 (0%)	
Respiratory, *n* (%)	1 (11.1%)	6 (9.5%)	0 (0%)	
Digestive, *n* (%)	1 (11.1%)	1 (1.6%)	0 (0%)	
Neurological, *n* (%)	0 (0%)	9 (14.3%)	1 (14.3%)	
Metabolic, *n* (%)	0 (0%)	5 (7.9%)	0 (0%)	
Sepsis, *n* (%)	1 (11.1%)	3 (4.8%)	3 (42.9%)	
Abnormal body temperature, *n* (%)	3 (33.3%)	5 (7.9%)	2 (28.6%)	
Cardiac arrest, *n* (%)	1 (11.1%)	8 (12.7%)	1 (14.3%)	
Trauma, *n* (%)	0 (0%)	4 (6.3%)	0 (0%)	
Other, *n* (%)	0 (0%)	13 (20.6%)	0 (0%)	
Charlson comorbidity index	0.7 ± 1.3	1.2 ± 1.5	0.4 ± 0.8	0.26
Blood biochemistry on admission				
CRP, mg/dl	0.3 (2.0)	0.4 (3.8)	0 (6.2)	0.35
Alb, mg/dl	3.4 ± 1.0	3.4 ± 0.8	3.3 ± 0.7	0.89
BUN, mg/dl	23.0 (10.5)	18.0 (12.8)	18.0 (13.3)	0.28
Cr, mg/dl	1.2 (0.8)	1.0 (0.7)	1.5 (1.2)	0.21
Lactate, mmol/l	1.8 (2.7)	2.8 (3.2)	4.7 (2.1)	0.14
Blood count				
WBC, ×10^3^/mm^3^	11.7 ± 3.6	12.5 ± 8.2	7.5 ± 4.4	0.11
Hb, g/dl	12.0 ± 3.6	12.4 ± 3.1	12.4 ± 1.3	0.89
APACHE II score	19.8 ± 8.2	18.2 ± 7.1	25.1 ± 8.5	0.07
AKI, *n* (%)	5 (55.6)	33 (52.4)	5 (71.4)	0.63
Outcome				
Length of ICU stay, *n* (day)	4.0 (6.3)	5.0 (8.0)	6.0 (7.5)	0.76
14 day mortality, *n* (%)	0 (0%)	10 (15.9%)	4 (57.1%)	0.01
28 day mortality, *n* (%)	0 (0%)	10 (15.9%)	4 (57.1%)	0.01

Of those patients who were L-FABP negative at admission, 53 remained negative, five were weakly positive and one was strongly positive 6 h later. Of those patients who were weakly positive for L-FABP at admission, seven were negative, two remained weakly positive, and one was strongly positive 6 h later. Of those patients who were strongly positive at admission, one was negative, another improved weakly, and eight remaining strongly positive 6 h later (Table 2).

**Table 2.** Results of urinary L-FABP at admission and 6 h later.

Judgment	6 h later (*n* = 79)		
	Negative (*n* = 61)	Weakly positive (*n* = 8)	Strongly positive (*n* = 10)
At admission (*n* = 79)			
Negative (*n* = 59)	53	5	1
Weakly positive (*n* = 10)	7	2	1
Strongly positive (*n* = 10)	1	1	8

AKI: acute kidney injury; Alb: albumin; APACHE: acute physiology and chronic health evaluation; BUN: blood urea nitrogen; Cr: creatinine; CRP: C-reactive protein; Hb: hemoglobin; ICU: intensive care unit; WBC: white blood cell.

There was one patient who was categorized as weakly positive in the first judgment at T1 and categorized as negative a week later, while one patient was categorized as negative in the first judgment and classified as weakly positive a week later. All other judgments were matched. The ICC was 0.93 and the kappa coefficient was 0.94 (eTable 1, Supplemental File).

There was a significant difference in 14-day mortality amongst the three groups (decreased vs. unchanged vs. increased: 0 *vs.* 15.9 *vs.* 57.1%, *p* = 0.01). The 28-day mortality rate was also significantly associated with changes in urinary L-FABP levels (*p* = 0.01). However, there was no significant difference in the length of ICU stay (*p* = 0.76) (Table 1).

Univariate analysis showed that, urinary L-FABP variation (odds ratio [OR] = 8.285, 95% confidence interval [CI] = 1.798–38.166, *p* = 0.01), urinary L-FABP at 6 h later (odds ratio [OR] = 3.657, 95% CI = 1.729–7.736, *p* < 0.01), lactate (OR = 1.234, 95% CI = 1.060–1.437, *p* = 0.01), and APACHE II score (OR = 1.124, 95% CI = 1.023–1.235, *p* = 0.02) were significantly associated with 14-day mortality (Table 3).

**Table 3.** Univariate analysis.

Parameters	Odds ratio	95% CI	*p*-Value
Age	1.020	0.985–1.056	0.28
Male	2.292	0.582–9.027	0.24
Height	2.654	0.008–854.273	0.74
Weight	1.008	0.967–1.050	0.72
Body mass index	1.019	0.891–1.165	0.79
Primary disease	1.005	0.830–1.217	0.96
Charlson comorbidity index	1.113	0.776–1.598	0.56
CRP/Alb	1.033	0.885–1.205	0.68
BUN	1.005	0.986–1.024	0.63
Cr	1.180	0.670–2.078	0.57
Lactate	1.234	1.060–1.437	0.01
WBC	1.000	1.000–1.000	0.12
Hb	0.989	0.816–1.199	0.90
APACHE II score	1.124	1.023–1.235	0.02
AKI	3.781	0.965–14.822	0.06
L-FABP at admission	2.012	0.977–4.145	0.06
L-FABP at 6 h later	3.657	1.729–7.736	<0.01
L-FABP variation	8.285	1.798–38.166	0.01

AKI: acute kidney injury; Alb: albumin; APACHE: acute physiology and chronic health evaluation; BUN: blood urea nitrogen; Cr: creatinine; CRP: C-reactive protein; Hb: hemoglobin; L-FABP: liver-type fatty acid-binding protein; WBC: white blood cell.

Analysis of L-FABP at admission and 6 h later revealed that L-FABP 6 h later was associated with 14-day mortality (eTable 2, Supplemental File).

In the multivariate analysis, based on the urinary L-FABP variation, the unchanged group is about 16 times more likely to die than the decrease group, and the increased group is about 16 times more likely to die than the unchanged group (OR = 16.783, 95% CI = 1.817–154.989, *p* = 0.01). Moreover, lactate levels were significantly associated with 14-day mortality (OR = 1.278, 95% CI = 1.025–1.592, *p* = 0.03) (Table 4).

**Table 4.** Multivariate analysis.

Parameters	Odds ratio	95% CI	VIF	*p*-Value
Age	0.997	0.936–1.061	2.54	0.92
Male	9.863	0.735–132.411	1.32	0.08
Body mass index	0.871	0.721–1.053	1.40	0.15
Primary disease	1.043	0.763–1.426	1.44	0.79
Charlson comorbidity index	0.927	0.577–1.488	1.16	0.75
CRP/Alb	1.039	0.810–1.332	1.16	0.76
Lactate	1.278	1.025–1.592	1.23	0.03
APACHE II score	1.086	0.943–1.251	2.07	0.25
AKI	6.309	0.777–51.201	1.59	0.08
L-FABP variation	16.783	1.817–154.989	1.15	0.01

AKI: acute kidney injury; Alb: albumin; APACHE: acute physiology and chronic health evaluation; CRP: C-reactive protein; L-FABP: liver-type fatty acid-binding protein.

**Figure 2.**
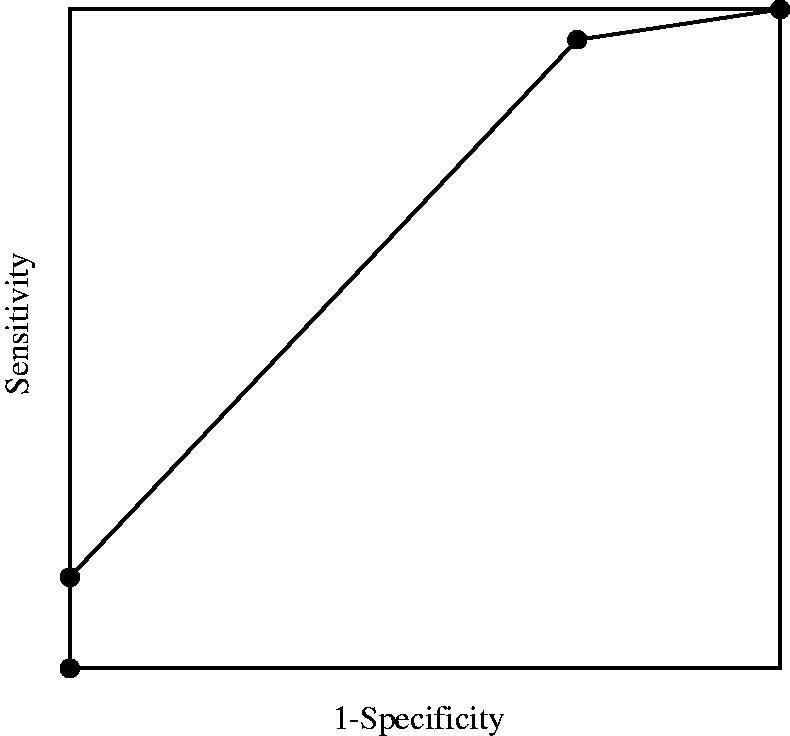
The receiver operating characteristic curve about L-FABP variation.

The distribution of propensity scores used for sensitivity analysis is shown in eTable 3 (Supplemental File). Sensitivity analysis showed that L-FABP variation was significantly associated with 14-day mortality along with propensity score (eTable 4, Supplemental File). The AUC of the ROC curve of L-FABP variation was 0.67 (Figure 2).

Finally, for additional information, eTable 5 shows a list of causes of death. Moreover, the results of urinary L-FABP levels (negative/positive) are shown in eTable 6 when patients were classified into non-survivors and survivors. In this study, weakly positives and strongly positives are collectively regarded as positive. The predictive values that were calculated from the table are as follows: negative predictive value at T1 was 84.7%, the positive predictive value was 25.0%, the negative predictive value at T2 was 91.8%, and the positive predictive value was 50.0%.

Among all patients, five required RRT.

## Discussion

In this study, we found that variation in urinary L-FABP levels at 6 h was significantly associated with 14-day mortality. Notably, L-FABP levels can be determined within 15 min using a semi-quantitative assay kit.

Urinary L-FABP is not only reported to be useful for the early diagnosis of AKI [2,3] but it has also been reported to be a useful prognostic factor [21,22]. In a previous study, Doi et al. reported that urinary L-FABP levels at admission are associated with the 14-day mortality rate in ICU patients [5]. The mechanism by which urinary L-FABP affects prognosis is yet to be elucidated but may be related to L-FABP being a marker that reflects systemic organ ischemia. Urinary L-FABP is considered a useful marker for renal ischemia [3–5], and in cases where urinary L-FABP is high, ischemia may occur in other organs. Thuijls et al. reported a link between increased urinary L-FABP levels and intestinal damage [8]. It has also been reported that urinary L-FABP levels increase in patients with post-traumatic sepsis and abdominal surgery [14]. Additionally, urinary L-FABP has been reported to be a marker for damage in each organ, especially ischemic injury, beyond being a conventional marker for predicting AKI. This can be explained by the fact that, in our findings, urinary L-FABP variation was associated with 14-day mortality, i.e., an increase in urinary L-FABP was associated with elevated mortality. The assumed theory behind this is that organ damage, including AKI, is indicated by an increase in urinary L-FABP, which improves within 6 h in patients with a good prognosis and worsens in those with a poor prognosis. As shown in Table 3 and eTable 2, it is important to observe not only L-FABP at admission but also its change at 6 h. The report by Doi et al. focused on the development of AKI and used quantitative urinary L-FABP levels that were measured upon admission [5]. In addition, while analyzing 14-day mortality as the objective variable, some factors related to prognosis, such as BMI, CRP, comorbidities other than diabetes, and lactate levels were not examined. In the present study, we examined the aforementioned factors. Furthermore, by examining variation in urinary L-FABP levels, it is possible to predict the course of disease that cannot be predicted by measuring urinary L-FABP levels at admission alone. In the present study, lactate was also found to be a significant factor, which is known to reflect organ ischemia and peripheral circulatory insufficiency, along with predicting short-term prognosis [20]. We evaluate ICU admission based on the vital signs of the patient, the severity of the disease, and the need for mechanical support; however, it is possible that lactate levels may also be used as a reference. Similar to lactate levels, urinary L-FABP may reflect peripheral circulatory dysfunction and organ ischemia. Furthermore, it is remarkable that no deaths were observed in the group with decreased urinary L-FABP levels. Urinary L-FABP variation may not have a high positive predictive value for death; however, it may have a high negative predictive value. The usefulness of patient management to reduce urinary L-FABP levels should be investigated in future studies.

In univariate analysis, WBC was close to a significant range, which may reflect the effects of physical stress that has some effect on mortality. None of the patients had blood disorders. The length of ICU stay did not affect prognosis, which may be because the ICU stay will be short if the patient dies shortly after admission.

Measuring urinary L-FABP levels requires testing after freezing the urine sample [3] and it usually takes several days to obtain the results. It is possible to obtain quantitative results within the same day if there are reagents and equipment for measuring urinary L-FABP in the hospital; however, it still takes several hours. Although quantitative testing has been used previously in a study conducted by Doi et al. [3], using this method, it is difficult to predict the prognosis within a few hours and respond clinically. However, the semi-quantitative kit used in the present study is highly convenient and time-efficient. Similar results may be obtained by quantifying urinary L-FABP levels. However, two-point measurements are required to determine variations, and convenience and speed enhance the superiority of this kit. The ability to predict prognosis rapidly results in the severity of patients being stratified in a short time, which is in turn considered to be useful for the appropriate distribution of medical care.

This study has some limitations. First, because this was a single-center, observational study, the sample size may be small and may not represent the entire population. The results of multivariate analysis are also not highly reliable; larger sample size studies are needed to confirm the findings. Moreover, the effects of unmeasured confounding factors have not been eliminated, therefore the results cannot be generalized in clinical practice. Second, we investigated only patients with indwelling bladder catheters, which may be selectively biased. Third, the judgment is made semi-quantitatively and lacks objectivity. However, intra-rater reliability is evaluated and it is considered to be an extremely reproducible evaluation item based on the ICC and Kappa coefficient. Fourth, the difference in infusion volume was not examined. Infusion sometimes causes dilution to urine, which can mask the detection of L-FABP. However, as a basic treatment policy, we optimize the intravascular volume by using vital signs, echo, and stroke volume variation as indicators. Therefore, the infusion solution may not significantly change the L-FABP results.

## Conclusions

Urinary L-FABP variation at 6 h after admission was significantly associated with 14-day mortality. Moreover, it may be easily and rapidly evaluated using a semi-quantitative assay kit.

## Supplementary Material

Supplemental Material

## Data Availability

The datasets used and/or analyzed during the current study are available from the corresponding author on reasonable request.
