# Supplementary material for: Urinary liver-type fatty acid-binding protein variation as a predictive value of short-term mortality in intensive care unit patients
Source: Ren Fail. 2021 Jun 30;43(1):1041–8. doi: 10.1080/0886022X.2021.1943439 (PMC8253184; doi:10.1080/0886022X.2021.1943439)
Supplement: Supplemental Material [file IRNF_A_1943439_SM6183.pdf]

eTable 1. Correspondence about match of judgment of urinary L-FABP on admission and kappa

**Judgement**		**A week later**		
		Negative	Weakly positive	Strongly positive
**The first judgement**	Negative	58	1	0
	Weakly positive	1	9	0
	Strongly positive	0	0	10

	Value	95%CI	P
ICC	0.93	0.086 – 0.952	N/A
Kappa	0.94	0.771 – 1.000	<0.01

CI, confidence interval; ICC, intraclass correlation coefficient; N/A, not applicable

eTable 2. Multivariate analysis about single time point measurement of L-FABP

Parameters	Odds ratio	95% CI	VIF	P value
Age	0.994	0.930 – 1.062	2.68	0.85
Male	7.947	0.780 – 80.927	1.33	0.08
Body mass index	0.852	0.695 – 1.045	1.45	0.12
Primary disease	0.922	0.620 – 1.371	1.61	0.69
Charlson Comorbidity Index	1.086	0.629 – 1.875	1.40	0.77
CRP/Alb	1.033	0.809 – 1.319	1.16	0.79
Lactate	1.219	0.969 – 1.534	1.47	0.09
APACHE II score	1.048	0.902 – 1.218	2.36	0.54
AKI	4.245	0.573 - 31.433	1.62	0.16
L-FABP at admission	0.209	0.036 – 1.196	2.56	0.08
L-FABP at 6h later	12.169	1.792 - 82.634	2.97	0.01

AKI, acute kidney injury; Alb, albumin; APACHE, acute physiology and chronic health evaluation; CI, confidence interval; CRP, C-reactive protein; L-FABP, liver-type fatty acid-binding protein; VIF, variance inflation factor

eTable 3. Propensity score distribution

Parameters	Decreased group (n = 9)	Unchanged group (n = 63)	Increased group (n = 7)	P value
Propensity score	0.11 (0.05, 0.4)	0.11 (0.03, 0.2)	0.24 (0.1, 0.3)	0.17

eTable 4. Logistic regression of sensitivity analysis

Parameters	Odds ratio	95% CI	P value
Propensity score	394.938	9.919 – 15724.430	<0.01
L-FABP variation	7.960	1.609 – 39.371	0.01

CI, confidence interval; L-FABP, liver-type fatty acid-binding protein

eTable 5. List of causes of death

Causes of deaths (n=14)
Cerebral hemorrhage （n=3, 21.4%）
Subarachnoid hemorrhage (n=1, 7.1%)
Trauma (n=1, 7.1%)
Sepsis (n=3, 21.4%)
Cirrhosis (n=1, 7.1%)
Interstitial pneumonia (n=1, 7.1%)
Cardiac arrest (n=4, 28.6%)

eTable 6. Distribution of judgment between non-survivors and survivors.

Judgement		Non-survivors (n=14)	Survivors (n=65)
**at admission (n=79)**	Negative, n (%)	9 (64.3%)	50 (76.9%)
	Positive, n (%)	5 (35.7%)	15 (23.1%)
**6h later (n=79)**	Negative, n (%)	5 (35.7%)	56 (86.2%)
	Positive, n (%)	9 (64.3%)	9 (13.8%)
